# Recent Advances in Attention Bias Modification for Substance Addictions

**DOI:** 10.3390/ijerph15040676

**Published:** 2018-04-04

**Authors:** Melvyn Weibin Zhang, Jiang Bo Ying, Guo Song, Daniel S. S. Fung, Helen E. Smith

**Affiliations:** 1 National Addictions Management Service, Institute of Mental Health, Singapore 539747, Singapore; song_guo@imh.com.sg; 2 Family Medicine and Primary Care, Lee Kong Chian School of Medicine, Nanyang Technological University, Singapore 66308232, Singapore; h.e.smith@ntu.edu.sg; 3 National Healthcare Group, National Psychiatry Residency Program, Singapore 539747, Singapore; yingjiangbo@gmail.com; 4 Department of Developmental Psychiatry, Institute of Mental Health, Singapore 539747, Singapore; daniel_fung@imh.com.sg

**Keywords:** attention bias, attention bias modification, substance abuse, addiction, E-Health, M-Health

## Abstract

Research on attentional bias modification has increased since 2014. A recent meta-analysis demonstrates evidence for bias modification for substance disorders, including alcohol and tobacco use disorders. Several pharmacological trials have shown that pharmacological agents can attenuate and modify such attentional bias. The pharmacological trials that have appeared to date have produced mixed results, which has clinical implications. Developments in Internet and mobile technologies have transformed how attention bias modification is currently being achieved. There remains great potential for further research that examines the efficacy of technology-aided attention bias interventions.

## 1. Overview of Prior Literature Review

Field et al. [1] and Cox et al. [2] have each highlighted the presence of attentional biases in substance use disorder and evaluated the clinical importance of attentional biases in their review and opinion articles. Attention biases are unconscious processes that typically cause individuals with substance use disorder to automatically orientate their attention towards substance-related cues in their naturalistic environment, with subsequent difficulty in disengaging from these cues [3]. Attentional biases are evaluated by a variety of measures. Most commonly, attentional biases are assessed using reaction time tasks, such as the Stroop task or the Visual Probe task [4]. In the Stroop task, participants are required to name the color of both the neutral and the drug-related word. The differences in the reaction time provide evidence for the presence of attentional biases. Similarly, for the visual probe task, participants are required to respond to a probe that replaces either a neutral or a substance-related image. The presence of attentional bias is demonstrated when participants demonstrate a faster reaction time responding to the probe that replace substance-related images [4].

Based on the dual-process model, it has been proposed that the chronic use of substances will increase the automatic processing of substance-related cues [5]. The dual-process model also proposed that these automatic tendencies tend to be coupled with inhibitions in normal cognitive control processes [5]. In a narrative review by Field et al. [1], attentional biases were of importance as they could predict the potential for relapse in individuals. However, the authors did not recommend that attentional bias modification should be included as part of the conventional treatment program, as the evidence about efficacy was mixed [1]. Instead, Field et al. [1] highlighted a need for larger clinical trials to be conducted to generate more conclusive evidence. They also recommended that future research should focus on individuals, who are at high risk of relapse. These individuals have higher temptation and motivation to use the drug, with attentional biases possibly being enhanced in this context.

While Field et al. [1] highlighted that there is mixed evidence for attentional bias, Cox et al. [2] reviewed several attentional bias programs, including the alcohol attention control training program (ACTP) [4]. They reported that the attentional bias program is efficacious for re-training attentional bias, reducing craving, and enhancing self-efficacy and perceived self-control in a sample of harmful drinkers. Cox et al. [2] also reported the efficacy of attentional bias modification for drug-related attentional bias, such as attentional bias to opiate cues, in a sample of drug abusers, who were undergoing methadone maintenance therapy. As described in Cox et al. [2], attentional re-training typically involves an intervention that helps to retrain bias away from substance-related cues. For example, the visual probe task would involve having probes to replace the neutral rather than the substance-related stimuli [6]. These prior studies based on evidence arising from individual studies conducted before 2014, have already demonstrated the potential of targeting attentional biases amongst substance use individuals.

## 2. Objectives of Current Perspective Article

Since 2014, there have been further advances in the field of experimental psychology, with more research conducted on attentional bias modification. Attentional bias in addictive disorders has now been extensively investigated in a variety of addiction disorders [6,7]. Thus, the main objective of the current perspective article is to provide an overview of the literature related to attentional bias modification for substance use disorders. Secondly, it reviews how technological advances have aided in the delivery of attentional bias modification interventions. Thirdly, it reviews the effect of pharmacological agents on attentional biases. Finally, it describes the authors’ perspectives on the clinical and research implications of attentional bias modification.

## 3. Existing Evidence for Attentional Bias Modification

To date, attentional bias modification interventions have been evaluated for a variety of addiction disorders, ranging from substance addiction to behavioral addiction. A prior meta-analytical study has synthesized the evidence for cognitive bias modification based on randomized trials published until 2015 [8]. The review examined 25 randomized controlled trials: 18 involved participants with alcohol use disorder and 7 involved participants with tobacco use disorder. In the review, the visual probe task was used in all twelve studies that specifically targeted attentional biases [8]. It should be noted that the meta-analysis objective was to review cognitive bias modification; hence, the authors have included other studies that targeted other biases, such as approach biases and interpretative biases [8]. Nevertheless, the prior review reported that cognitive bias modification was effective in the reduction of cognitive bias (Hedges’ G = 0.60) [8]. However, there does not appear to be a relationship between the reduction in bias and the consequential reduction in cravings, which are thought to lead to the improvements in addiction outcomes [8]. Cristea et al. have provided two reasons as to why there was no association between bias reduction and improvements in craving. Firstly, it is possible that bias modification has effectively reduced bias, but this reduction in biases did not translate directly to affecting the objective clinical outcome. Secondly, it could be that more time is needed for the changes in biases to be reflected as changes in symptoms [8]. Jones et al. [9] undertook a review of all currently published meta-analyses on cognitive bias and reported that cognitive bias modification programs consistently targeted bias with effect sizes of 0.52–0.81. This review highlighted that the long-term efficacy of cognitive bias modification was only evident in studies with participants that have a substance addiction disorder [9]. Impulsivity has been proposed to be a mediator of attentional bias, and this has been examined in a prior meta-analytic study by Coskunipar and Cyders [10]. In the prior meta-analysis, a total of 13 studies that explored the relationship between substance-related attentional bias and impulsivity were analyzed. A significant but small effect size was found between that of impulsivity and attentional bias [10]. The presence of impulsivity would cause individuals to have heightened attentional biases [10].

Apart from the existence of further studies evaluating the effectiveness of bias modification, the advances in experimental psychology has also led to the creation of newer methods for bias assessment. Leeman et al. [4] highlighted newer approaches in their critical review, such as eye movement tracking. This has been successfully utilized in other studies for the assessment of biases [11,12]. Apart from eye movement tracking, electrophysiological measures have been used in the assessment and the characterization of the changes following attentional bias modification. In their recent study, Zhao et al. [3] collated electrophysiological data from individuals abstaining from taking opiates, who were administered a dot–probe task. Their results demonstrated that abstaining individuals still had biases towards substance-related cues, with electrophysiological data suggesting that there was an overall shorter P1 latency, larger N1, N2, and P2 amplitudes, and a shorter latency and amplitude for P3 [3]. The data from these electrophysiological measures demonstrate the increased involvement in certain brain regions, such as the dorsal posterior cingulate cortex, superior parietal lobule, and inferior frontal gyrus [3]. In their study using electrophysiological measures, Pravaz et al. [13] also demonstrate that there were changes in the late positive potential as attentional bias was modified. Recent fMRI studies have also demonstrated that attentional bias is associated with activation in the following regions of the anterior cingulate cortex, dorsolateral prefrontal cortex, insula, nucleus accumbens, and amygdala [14,15,16].

In summary, the current evidence highlights that attentional biases are present in substance addictions. These attentional biases are amenable to modification using conventional interventions for attentional bias modification. While attentional biases were reduced following bias modification, the reduction in attention bias was not associated with improvement in other symptoms, such as cravings. The advances in the methods of assessing attentional biases and analyzing the involvement of neural circuitry have several implications for further research. 

## 4. Pharmacological Drugs on Attentional Bias

Dopaminergic neurotransmission has also been responsible for unconscious attentional processes. In their systematic review, Luijten et al. [17] synthesized the evidence-related pharmacological intervention for attentional bias in substance use disorders, which includes stimulant, opioid, and cocaine-dependent individuals. The evidence showed that medications that modulate dopamine do affect the strength of attentional biases [17]. In the studies included in this systematic review, the pharmacological agents used were amisulpride (D2/D3 receptor antagonist), pramipexole dihydrochloride (D2/D3 receptor agonist), haloperidol (D2/D3 receptor antagonist), methylphenidate (dopamine reuptake inhibitor), D-cycloserine (partial glycine-site NMDA agonist), and acute tyrosine or phenylamine depletion. The measurements of attentional biases were conducted using conventional paradigms, such as the dot–probe task or the Stroop task. There was a reduction in attentional biases after the acute administration of these pharmacological agents [18]. However, these findings are confined to acute effects following a single dose of a particular medication. There were no longer-term studies conducted to demonstrate that attentional bias would diminish following longer-term administration of dopamine modulating agents [17]. 

While the dopaminergic neurotransmission potentially could account for these unconscious attentional processes, other neurotransmitter systems have been implicated, such as the noradrenaline system and the glutaminergic neurotransmission system. Atomoxetine [19], a pharmacological agent modulating noradrenaline reuptake, has been shown to be efficacious in reducing attentional biases to cocaine-related cues. It is postulated that the administration of atomoxetine increases noradrenaline levels, particularly in the prefrontal cortex. The increase in the noradrenaline levels is associated with increased conscious inhibitory control. Despite the promising findings, it should be noted that the study evaluated only the acute effects of a single dose of atomoxetine. 

Liu et al. [20] evaluated the effect of the selective serotonin reuptake inhibitor of escitalopram on attentional bias in a sample of cocaine-dependent individuals. The study by Liu et al. was based on the evidence generated from a preclinical study, which suggests that agonists of the 5-HT2C receptor could affect attentional biases [18]. The dosage of escitalopram administered was in the range of 10–20 mg and subjects were followed up for a total of 28 days [20]. The selective serotonin reuptake inhibitor has been shown to be effective in reducing attentional biases only in the acute phase, which was determined by the reduction in attentional bias from baseline that occurred five hours following the administration [20]. The continued administration of escitalopram did not produce any significant changes in attentional biases from baseline when Stroop testing was re-administered subsequently. To our knowledge, Liu et al. [20] is the only study to date that has administrated a course of the pharmacological agent over an appropriate follow-up period. The acute effects of escitalopram on attentional biases might be due to the increased activation of the 5-HT2C receptors, while the lack of a reduction in attentional biases following chronic administration might be accounted for by adaptive changes in post-synaptic receptors [20]. Liu et al. [20] also pointed out that the chronic administration of escitalopram might have resulted in desensitization of the 5-HT1A auto receptors; hence, the prolonged administration of escitalopram did not result in further increases of serotonin levels. 

The findings from these studies demonstrate that pharmacological agents are helpful in the modulation of attentional biases when these medications are given acutely. No studies have determined whether there will be further reduction in attentional biases if dopaminergic or other medications are administered for a longer duration. The evidence arising from Liu et al. demonstrates that the chronic administration of serotonergic medications does not alter attentional biases. There are clinical and research implications arising from these results, which will be discussed subsequently. The findings from the current studies also highlight that researchers seeking to evaluate attentional bias and the effectiveness of bias modifications need to consider the pharmacological agents that the sample individuals are already on, as it might confound the results of the psychological intervention. 

## 5. E-Health & M-Health Based Attentional Bias Modification Interventions

The introduction of digital technologies into healthcare has revolutionized various healthcare practices. E-health refers to the use of Internet-based technologies, telephone, and text-based messaging to support health interventions [21]. M-health refers to how smartphone and their accompanying applications are used as health care interventions [21]. Such technologies are being used in medical and surgical disciplines. In the latter setting, these technologies have changed the delivery of interventions for attentional bias modification [22]. Conventionally, attentional bias modification interventions, which involves training individuals to gain control over their automatic responses, were delivered in a controlled environment, such as a laboratory. Nowadays, interventions can be delivered remotely. In their study, Wiers et al. [22] evaluated an automated cognitive bias modification program for problem drinkers that was delivered using the Internet. In this study, the approach and avoidance biases were targeted as part of their online cognitive bias intervention. Approach and avoidance biases are similar to that of attentional bias as they are all unconscious processes and tend to cause individuals to experience a lapse or relapse [22]. Wiers et al. observed a reduction in drinking across all the groups, including the group that has received only control training [16]. Despite the findings of the study, Wiers et al. showed the potential of delivering attentional bias modification programs using the Internet [22]. However, one of the main limitations of such a mechanism of delivery would be that of attrition [22]. In the study conducted by Wiers et al., it should be noted that half of the recruited participants were not commenced on the intervention and another half of the sample dropped out during the intervention [22]. 

Web-based attentional bias modification programs have also been evaluated in adolescents with anxiety and depression, with demonstrable efficacy of the web-based program in reducing both anxiety and depressive symptoms [23]. The study also reported high attrition rates from their web-based interventions [23]. To tackle attrition, other researchers have introduced gamification elements into existing conventional attention bias modification tasks. In their recent study, Boendermaker et al. [24] described how they introduced serious game elements into a conventional visual probe modification task for individuals with alcohol use disorder. In their design, they modified the conventional visual probe task to resemble that of a slot machine game, including in-game credits and rewards [24]. Despite the introduction of gamification elements, it did not affect attrition and did not increase the motivation in participants to complete the tasks, as participants in all training conditions felt that the tasks became boring over time. In a prior study by Boendermaker et al. [25], gamified version of cognitive retraining was compared to that of placebo and regular training in a sample of participants with alcohol use problems. The authors reported that despite there being no changes in bias or drinking behavior, the addition of gaming elements did help to increase the motivation to train. 

Other studies have also described how smartphone devices have been utilized in the delivery of attentional bias modification intervention [26,27]. In the study by Clarke et al., a smartphone device was used to deliver attention bias modification for individuals suffering from insomnia. They reported that bias modification was helpful in reducing the arousal symptoms and improving the overall sleep quality [26]. Yang et al. [27] reported the feasibility of delivering cognitive bias modification for individuals with social anxiety disorder using smartphone device. Hence, it is clear from the existing literature that technological advances are increasingly incorporated in interventions for attentional bias modification, although there needs to be further work to determine the efficacy of these interventions. The fact that gamification could help to increase motivation for training in some attention bias interventions does imply that this remains an area for further research. 

## 6. Clinical and Research Implications

There are clinical and research implications that arise from recent advances in attentional bias modification. The findings from the meta-analysis demonstrate that attentional biases could be subjected to modification. Targeting attentional biases is pertinent as according to the dual-process theoretical model, the repeated usage of an addictive substance would increase the automatic processing of substance-related cues, including attentional biases and automatic approach tendencies for substance-related cues [1]. Furthermore, these automatic processes are thought to suppress the normal cognitive control processes [1]. Targeting attentional biases help to address one of the key factors that could result in individuals having a lapse or relapse. Furthermore, these biases are not typically targeted in conventional psychological therapies, such as cognitive behavioral therapy. Based on the review by Cristea et al., it appears that attentional bias modification has been well-studied for alcohol and tobacco use disorders. Even though this review searched for trials involving other substance use disorders, such as cannabis, opiate, and amphetamine, it appears that no randomized trials were found for their synthesis. Hence, this warrants further review to synthesize the evidence for attentional bias and evaluate the effectiveness of attention bias modification for other substance disorders. 

In our literature review, we have not managed to identify studies involving bias modification in individuals with dual diagnoses. However, in the clinical context, it is common for individuals to present with comorbidities or with dual diagnoses. To date, researchers have largely focused on samples with an isolated diagnosis or disorder. It is important for there to be further research evaluating the efficacy of attentional bias modification for individuals with dual diagnoses and comorbidities. 

The advances in assessment of attentional biases also imply that researchers are no longer confined to a single measure, which was previously reaction time, in the determination of attentional biases. More recently, electrophysiological measures have been used to demonstrate the presence of attentional biases. Thus, it is pertinent for researchers who are planning further studies in this area to consider both an indirect and direct measure, which can potentially improve the accuracy of detection of attentional biases. Direct measures, such as electrophysiological measures, are ideal for demonstrating the neural circuits that are affected and potentially altered using bias modification. Direct measures could also inform clinicians of real-time changes in neural circuitry or changes in brain activation following each administration of bias modification. It would be crucial for future research to examine how technology-aided delivery of an attention bias modification task could be coupled with another direct objective measure, such as electrophysiological measurements. 

Furthermore, given how repetitive the task might be, it will be wise to consider pairing attentional bias modification intervention with motivational interventions [28]. In the protocol by Boffo et al., the authors have suggested the inclusion of motivational interviewing, as it is believed that motivational interviewing could help to support the individual in making changes to his/her behavior [28]. In addition, there appear to be mixed findings arising from pioneering work involving gamification for attention bias intervention. To some extent, this might imply that there remains a need for some elements of face-to-face contact, but it also implies that there needs to be further research to select the most appropriate gamification strategy for attention bias interventions. 

More research is needed to evaluate web-based interventions to establish their efficacy. There are advantages to web-based interventions, as individuals would not need to be confined to a particular environment to receive the interventions for attentional bias modification. Moreover, the advances in technology might also allow for users to partake in these interventions immediately when they are at risk of relapse.

The findings from the pharmacological trials to date show that pharmacological agents are only efficacious in the acute reduction of attentional biases. While a diverse range of pharmacological agents has been evaluated, there remains a need for further studies evaluating the longer-term efficacy of pharmacological agents that modulate dopaminergic and other neurotransmitters. Current findings are important clinically as if these agents are useful in the chronic reduction of attentional biases, they could be considered for usage to prevent lapse and relapse in individuals. However, the usage of these pharmacological agents needs to be carefully considered due to their high propensity for inducing side effects. This includes extra-pyramidal side effects, amotivation, and apathy for medications, such as haloperidol. To minimize these side effects, it would be ideal if there were similarly efficacious psychological interventions for attention bias modification for which participants could be recruited. Researchers do need to take into careful consideration the pharmacological agents that individuals are on when they are evaluating attentional bias interventions, as some of these medications are potential confounders. 

## 7. Conclusions

In conclusion, there remains a need for further research synthesizing the evidence and effectiveness of attention bias modification for other substance disorders. It is also important for future research to evaluate the efficacy of attentional bias modification for individuals with dual diagnoses. Future research should also consider pairing an indirect measure with a direct measure for the assessment of biases. Motivational interventions and gamification strategies need further evaluation to determine their effectiveness in helping to increase intrinsic motivation of participants undertaking bias modification interventions. Studies should also evaluate web-based bias interventions to determine their efficacy. Future pharmacological trials should evaluate whether chronic administration of pharmacological agents is effective in reducing attentional biases.
